# A placebo-controlled study of the effects of ayahuasca, set and setting on mental health of participants in ayahuasca group retreats

**DOI:** 10.1007/s00213-021-05817-8

**Published:** 2021-03-10

**Authors:** M. V. Uthaug, N. L. Mason, S. W. Toennes, J. T. Reckweg, E. B. de Sousa Fernandes Perna, K. P. C. Kuypers, K. van Oorsouw, J. Riba, J. G. Ramaekers

**Affiliations:** 1 Department of Neuropsychology and Psychopharmacology, Faculty of Psychology and Neuroscience, Maastricht University, Maastricht, The Netherlands; 2 Institute of Legal Medicine, Goethe University of Frankfurt, Frankfurt, Germany; 3 Department of Clinical Sciences, Faculty of Psychology and Neuroscience, Maastricht University, Maastricht, The Netherlands

**Keywords:** Ayahuasca, Placebo, Field study, Empathy, Affect

## Abstract

Ayahuasca is a plant concoction containing *N,N*-dimethyltryptamine (DMT) and certain β-carboline alkaloids from South America. Previous research in naturalistic settings has suggested that ingestion of ayahuasca can improve mental health and well-being; however, these studies were not placebo controlled and did not control for the possibility of expectation bias. This naturalistic observational study was designed to assess whether mental health changes were produced by ayahuasca or by set and setting. Assessments were made pre- and post-ayahuasca sessions in 30 experienced participants of ayahuasca retreats hosted in the Netherlands, Spain, and Germany. Participants consumed ayahuasca (*N* = 14) or placebo (*N* = 16). Analysis revealed a main effect of time on symptoms of depression, anxiety, and stress. Compared to baseline, symptoms reduced in both groups after the ceremony, independent of treatment. There was a main treatment × time interaction on implicit emotional empathy, indicating that ayahuasca increased emotional empathy to negative stimuli. The current findings suggest that improvements in mental health of participants of ayahuasca ceremonies can be driven by non-pharmacological factors that constitute a placebo response but also by pharmacological factors that are related to the use of ayahuasca. These findings stress the importance of placebo-controlled designs in psychedelic research and the need to further explore the contribution of non-pharmacological factors to the psychedelic experience.

**Supplementary Information:**

The online version contains supplementary material available at 10.1007/s00213-021-05817-8.

## Introduction

Ayahuasca is a plant concoction originally used by shamans in the Amazon region for communication with spirits, magical experiences, rites of initiation, and healing rituals. This practice is commonly referred to as “shamanism” (Brito et al. 1996; Krippner 2000; McKenna 2004; Townsend 2004). The concoction is prepared by cooking leafs from the *Psychotria viridis* bush mixed with the liana *Banisteriopsis caapi*. These respectively contain the serotonergic 2A receptor agonist *N,N*-dimethyltryptamine (DMT), and β-carboline alkaloids such as harmine, harmaline, and tetrahydroharmine. Of note, the β-carboline alkaloids function as monoamine oxidase inhibitors (MAOI) allowing DMT to reach the central nervous system for a prolonged period of time. This leads to intense alterations in perception and sensory integration and the induction of a highly altered state of consciousness (McKenna 2004; Palhano-Fontes et al. 2015; Riba et al. 2001; Tupper 2008). The principles and techniques of traditional shamanic rituals where ayahuasca has been used historically have recently spread beyond its native habitat and have subsequently become adopted by the Western contemporary society. At present, ayahuasca is sought after by an increasing number of Westerners for various reasons such as “spiritual enlightenment,” “self-actualization,” “mystical experiences,” and “psychotherapy” (Fotiou 2012; Uthaug et al. 2018; Winkelman 2005). The prevalence of ayahuasca use worldwide as well as its (anecdotal) beneficial effects on mental well-being sparked scientific interest in its therapeutic potential (Barbosa et al. 2009; Barbosa et al. 2005; Barbosa et al. 2012; Dos Santos et al. 2016; Frecska et al. 2016). This has led to clinical, open-label (Sanches et al. 2016), and placebo controlled (Palhano-Fontes et al. 2019) studies that demonstrated a rapid antidepressant action of ayahuasca in patients with treatment-resistant depression. Yet, despite these promising findings, ayahuasca has not yet been developed into a regular medicine for the treatment of depression.

The use of ayahuasca as a treatment in non-controlled and non-clinical settings, i.e., ayahuasca retreats, has however become increasingly popular. A number of research groups have visited such ayahuasca retreats in order to conduct naturalistic “field” studies to determine whether the use of ayahuasca in non-clinical settings is also associated with improvements in mental functioning. Specifically, such observational studies have indicated that in healthy individuals, the use of ayahuasca is related to acute enhancement of flexible thinking (Kuypers et al. 2016), improvement in affect and cognition (Uthaug et al. 2018), and increased mindfulness-related capacities (Soler et al. 2016). In people with mental health problems, the use of ayahuasca in naturalistic settings has been associated with the recovery from eating disorders (Lafrance et al. 2017) and the enhancement of emotion regulation in individuals with borderline-like traits (Domínguez-Clavé et al. 2018).

Nevertheless, none of these explorative field studies have controlled for the placebo effect, thereby introducing the possibility that changes observed in these studies could be attributed to factors other than the pharmacological agent ayahuasca. Placebo effects can be very strong. For example, in a randomized clinical trial that demonstrated superiority of ayahuasca over placebo in the treatment of depression, a response rate of 46% and 26% was observed at 1 and 7 days after treatment in the placebo group (Palhano-Fontes et al. 2019). In another study in which participants expected to receive psilocybin but actually consumed a placebo, the majority (61%) reported to experience some drug effect (Olson et al. 2020). Individual variation in the placebo response however was high. Many participants reported no changes while others reported moderate to strong effects (Olson et al. 2020). Non-pharmacological factors that have been recognized to have an impact on the behavioral and psychological effects of psychedelics include set and setting (Hartogsohn 2016). Set refers to the intentions, mood state, and expectations of the individual partaking in an ayahuasca ritual, while setting refers to the context in which the ceremony takes place including all sensory modes (e.g., auditory, music; visual, tactile), social environment (e.g., being alone or in a group, in nature or in a building, presence of a leader), as well as the set of those present in a ceremony that surround an individual (Haijen et al. 2018; Hartogsohn 2014, 2016, 2017; Lawn et al. 2017; Leary et al. 1963; Shewan et al. 2000). Individual intentions and expectations of healing play a prominent role in ayahuasca sessions as these are made explicit in group discussions prior to drinking ayahuasca, and these are similarly directed after the experience during integrative group sessions (Adamson and Metzner 1988). Moreover, group dynamics before, during, and after ceremonies with psychedelics in general are controlled and guided by facilitators or hosts who aim to maximize the setting in which the session takes place (Adamson and Metzner 1988; Blewett 1970; Trope et al. 2019). Of note, this may make the participant prone to the effects of suggestibility which might amplify the subjective effects (Carhart-Harris et al. 2015).

In order to understand the role of set and setting on psychological effects observed after participation in a non-clinical ayahuasca ceremony, a naturalistic, placebo-controlled, observational study was set up to address this knowledge gap. The primary objective of the present study was to assess differences in responses to ayahuasca and placebo in participants of naturalistic ayahuasca ceremonies. We hypothesized that set and setting would impact both groups, whereas pharmacological effects would only be observable in the ayahuasca group.

## Methods

We visited 6 ayahuasca retreats, hosted by a single organization, all taking place at several locations in Europe (the Netherlands, Spain, and Germany). The ayahuasca ceremonies were all structured in the same way. The organizers promoted the dates of their ayahuasca ceremonies online and attracted on average 15–25 ceremony participants per event. Most of them were one-time visitors with no or limited previous experience with ayahuasca. A small number of ceremony participants (up to *N*=6) per event were more experienced, and “students of an ayahuasca school” linked to the host organization. These individuals were in training to become facilitators of ayahuasca ceremonies.

The “students” exposed themselves repeatedly to ayahuasca ceremonies of the host organization. Most of the time, they drank ayahuasca while participating, but on some occasions, they were requested by the organizers to not drink ayahuasca in order to be able to observe the ceremony from the perspective of a facilitator. The host organization prepared freeze-dried ayahuasca and placebo capsules as part of their training program for “students.” Only “students of the ayahuasca school” were invited to participate in the present single-blind, placebo-controlled study, in which the organizers offered ayahuasca or an ayahuasca-placebo. If they consented to become a study participant, they received capsules containing either freeze-dried ayahuasca or a placebo substance during the ayahuasca ceremony, except for one retreat location where they consented to drink ayahuasca brew or placebo. Ceremony participants, who were not participating in the present study, drank ayahuasca brew. The substances were provided and administered by the ceremony organizers.

Study participants were invited to enter the study after they registered at the retreat. Inclusion criteria to participate included fluency in English, aged over 18, and written informed consent. Participation was voluntary, and no incentives to participate were provided. After inclusion, study participants completed a 30-min test battery prior to the ayahuasca session, which served as the baseline measurement, and in the morning of the next day, after the ceremony. This study was approved by the Ethics Review Committee Psychology and Neuroscience (ERCPN-175-03-2017) at Maastricht University, the Netherlands. All methods were carried out in accordance with relevant guidelines and regulations. Ayahuasca ceremonies were initiated and supervised by the host organization. The research team was not involved in the organization of the ceremonies or the production and administration of ayahuasca. Their presence was only observational.

### Dose administration

Study participants were randomly assigned by the host organization to receive either ayahuasca (*N* = 14) or placebo (*N* = 16) from one of the facilitators of the retreat and in the presence of a member of the research team. Additionally, both the facilitators that were present during the administration of ayahuasca and the subsequent ceremony, as well as the study participants, were blind to the actual treatments. Investigators, study participants, and facilitators were debriefed the next day, after the test session, about the content of the capsules that were administered. Study participants received 7 capsules with the option of taking 3 additional ones as a booster, after about 2 h of the first dose. A dose of 7 capsules was portrayed by the host organization as similar to as regular volume of ayahuasca brew. These capsules contained either freeze-dried ayahuasca or a placebo mixture that contained a mix of the following ingredients: coco powder, vitamins (unspecified), turmeric powder, quinoa, traces of coffee, and potato flour. Capsules were produced by the host organization. No information was available on the production, content, and storage of the capsules. Three ayahuasca capsules were collected in order to determine the concentration of alkaloids afterwards.

During one of the ceremonies, no capsules were available because they were not provided by the host organization to the site where which this ceremony took place. Instead a drink mixture was administered which was either regular ayahuasca tea or a placebo drink including coffee, coco powder, and balsamic vinegar; the latter was added to better mimic the authentic ayahuasca taste.

### Ayahuasca

The alkaloid concentrations in the ayahuasca capsules were determined after dissolution in 25 mL of water using high-performance liquid chromatography-electrospray ionization-time-of-flight mass spectrometry (HPLC-TOF MS) which was calibrated with pure reference substances of *N*,*N*-dimethyltryptamine (DMT; Cerilliant, Round Rock, TX, USA), harmine, and harmaline (Aldrich Chemistry, St. Louis, MO, USA). Weight of ayahuasca capsules and concentrations of DMT and harmalines are shown in Table 2. Doses per individual subject are shown in Table 1.

**Table 1:** Summary of the number of capsules per participant as well as the doses of DMT, harmine, and harmaline; the level of ego dissolution; and the individual and total (correct) treatment guesses of the facilitators and participants (PP)

Ayahuasca group								Placebo group				
PP	Capsules (#)	DMT (mg)	Harmine (mg)	Harmaline (mg)	EDI (%)	Correct guess facilitator	Correct guess participant	PP	Capsules (#)	EDI (%)	Correct guess facilitator	Correct guess participant
4	7	14.1	39.0	2.7	7.7	No	No	1	10	7.3	No	No
5	10	20.1	55.7	3.9	14.8	Yes	Yes	2	10	5.2	Yes	Yes
6	7	14.1	55.7	2.7	36.2	No	No	3	10	6.2	Yes	No
9	1 cup	NA	NA	NA	36.9	No	No	7	2 cups	59.4	Yes	No
10	1 cup	NA	NA	NA	40.0	Yes	Yes	8	2 cups	69.0	Yes	Yes
12	10	20.1	55.7	3.9	93.8	No	Yes	11	10	–	Yes	Yes
15	10	20.1	55.7	3.9	0.0	No	No	13	7	21.5	Yes	Yes
16	10	20.1	55.7	3.9	20.0	No	No	14	10	–	Yes	Yes
19	10	20.1	55.7	3.9	36.3	-	Yes	17	10	59.0	Yes	No
21	10	20.1	55.7	3.9	12.5	Yes	Yes	18	10	66.1	-	Yes
22	10	20.1	55.7	3.9	44.3	Yes	Yes	20	7	71.6	Yes	Yes
23	10	20.1	55.7	3.9	46.3	Yes	Yes	24	10	17.9	Yes	Yes
26	10	20.1	55.7	3.9	17.4	No	Yes	25	7	2.5	Yes	Yes
29	7	14.1	39.0	2,7	47.5	No	No	27	10	20.0	Yes	No
								28	10	21.3	yes	yes
								30	10	2.4	yes	yes

*NA* not analyzed

**Table 2:** Weights of ayahuasca capsules and their ingredients, concentrations, and doses of DMT, harmine, and harmalines per capsule

	Capsule weight	Ingredients	DMT	Harmine	Harmaline
	(mg)	(mg)	(mg/g)	(mg/g)	(mg/g)
Capsule 1	579.6	475.9	3.7	10.6	0.7
Capsule 2	703.8	599.6	3.8	10.5	0.7
Capsule 3	679.4	579.2	3.4	9.2	0.6
Mean±SD	654.3±65.8	551.6±66.3	3.6±0.2	10.1±0.8	0.7±0.1

### Setting of the retreat

After the registration, all ceremony participants were welcomed to the room where the session would start around midnight. The room had a mattress on the floor for each of the ceremony participants. There was also a plastic bucket available for each of the ceremony participants in case purging occurred. During the session, at least 2 facilitators from the host organization were present in the room. None of the investigators were present during the ayahuasca ceremony.

Throughout the session, the facilitators were sitting in front of the room guiding the session (playing music, singing, or giving instructions) while also being ready and alert to give individuals support if needed. A member of the host organization was present at the beginning of the session and distributed the capsules or drink to the study participants to ensure that neither the facilitator nor the participant knew which student was assigned to which condition (ayahuasca or placebo). Finally, once the session reached its completion in the early morning hours the following day, the study participants would go to sleep in the session room or in their dorms.

### Test battery

The test battery consisted of a demographic section, the multifaceted empathy test (MET), and five questionnaires: the Ego Dissolution Inventory (EDI); the 5-Dimensional Altered States of Consciousness Rating Scale (5D-ASC); the Depression, Anxiety, and Stress Scale 21 (DASS-21); the Brief Symptom Inventory 18 (BSI-18); and the Five Facets Mindfulness Questionnaire (FFMQ-39). All the material was provided in English. All the measures were filled out twice, i.e., at baseline and post-session except for the EDI and the 5D-ASC that was only filled out once, post-session, to assess the psychedelic experience in retrospect.

### Empathy

#### Multifaceted empathy test

The MET consists of 40 pictures of people in various emotional states, with 50% being positive and 50% negative (Dziobek et al. 2008). To assess cognitive empathy (CE), participants were asked to select the emotion word, out of four words, that matched the depicted emotion. To assess emotional empathy (EE), participants were asked to rate on a scale from 1 to 9 “how aroused does this picture make you feel” (implicit EE) and “how concerned do you feel for this person” (explicit EE). Implicit EE and explicit EE ratings per valence (positive and negative) were used as dependent variables. Previous validity and reliability analysis of the MET have shown to be in the good to highly satisfactory range (Dziobek et al. 2008), and previous studies have found it to be sensitive to the effects of psychedelics (Hysek et al. 2013; Kuypers et al. 2014; Kuypers et al. 2017a; Mason et al. 2019; Pokorny et al. 2017; Preller et al. 2015).

### The psychedelic experience

#### Ego Dissolution Inventory

The EDI is an 8-item self-report scale that assesses the participant’s experience of ego dissolution, with excellent internal consistency (Cronbach’s alpha = .93) (Nour et al. 2016). The participants score their experience by making a mark on a line that ranged from “No, not more than usual” (0 %) to “Yes I experience this completely/entirely” (100 %). The total EDI is scored by calculating the mean percentage of all the 8 items and ranges between 0 and 100%. The higher the total score, the stronger the experience of ego dissolution.

#### 5-Dimensional Altered States of Consciousness Rating Scale

The 5D-ASC is a 94-item self-report scale that assesses the participants’ alterations from normal waking consciousness with a Cronbach’s alpha range between 0.88 and 0.95 (Dittrich 1998; Dittrich et al. 2010; Studerus et al. 2010). The participant is asked to make a vertical mark on the line below each statement to rate to what extent the statements applied to their experience in retrospect (i.e., from 0 “No, not more than usually” to 100% “Yes, more than usually”), and the score ranges from 0 to 100%. The 5D-ASC measures 11 subscales; *experience of unity spiritual experience*, *blissful state*, *insightfulness*, *disembodiment*, *impaired control and cognition*, *anxiety*, *complex imagery*, *elementary imagery*, *audio-visual synesthesia*, *and changed meaning of perception.* Moreover, the 5D-ASC measures 5 key-dimensions which include oceanic boundlessness that identifies mystical-type experiences and has been compared with the “heaven” aspect of Huxley’s mescaline account (Dittrich 1998), anxious ego dissolution, visual restructuralization, auditory alterations, and reduction of vigilance*.*

### Subjective effects

#### Depression, Anxiety, and Stress Scale 21

The DASS-21 is the shorter version of the original self-report questionnaire Depression, Anxiety, and Stress Scale 42 with a Cronbach’s alpha of 0.93 (Henry and Crawford 2005). The purpose of the DASS-21 scale is to measure constructs of depression, anxiety, and stress ranging from 0 (normal) to 42 (extremely severe). The participants responded by rating the concordance with each statement from 0 “Did not apply to me at all” to 3 “Applied to me very much, or most of the time.” The subscale scores for depression *(α* = .88) with a range from normal = 0 to extreme severe = 28+, anxiety (*α* =.82) with a range from normal = 0 to extremely severe = 20+, and stress (*α* =.90) with a range from normal = 0 to extreme severe = 34+ are calculated by summing the scores for the items comprising the characteristic being measured (Henry and Crawford 2005). As the original DASS has 42 questions, the sum of the DASS-21 is multiplied by 2 to ascertain the comparable scores.

#### Brief Symptom Inventory 18

The BSI-18 is a self-report scale which contains subscales on somatization, depression, and anxiety (Zabora et al. 2001). Participants were asked to rate a list of issues people can experience on a 5-point Likert scale ranging from 0 “None at all” to 4 “Extremely.” Cronbach’s alpha (*α*) of the BSI subscales somatization, depression, and anxiety were .82, .87, and .84, respectively (Franke et al. 2017), suggesting strong internal consistency. BSI scores range from 0 to 24.

#### Five Facets Mindfulness Questionnaire 15

The FFMQ-15 is a 15-item self-report questionnaire which measures five different factors: (1) observe, noticing experiences that are both internal and external such as thoughts and emotions; (2) describe, describing internal experiences; (3) acting with awareness, focus on the present activity; (4) non-judgment, not evaluating or judging the present experience; and (5) non-reaction, allowing thoughts and feelings to come without acting or reacting upon them (Baer et al. 2006) (Gu et al. 2016). The purpose of this scale is to obtain an understanding of an individual’s mindfulness-related capacities. The participants answered the FFMQ by rating the concordance with each statement on a 5-point Likert scale that ranges from 1 “never true” to 5 “very often or always true.” The subscale scores are obtained by adding the relevant items for each of the five facets. Facet scores range from 8 to 40, except for the non-reactivity facet, which ranges from 7 to 35. The original scale has shown good internal consistency, and the Cronbach’s alpha (α) of each subscale was non-reaction = .77, non-judgment = .78, describe =.83, observe = .69, and awareness = .70 (Baer et al. 2006).

### Statistics

The statistical analysis was conducted in IBM SPSS Statistics 24 using a mixed model ANOVA that included a within subject factor time (two levels: baseline and post-session), a between subject factor treatment (two levels: ayahuasca or placebo), and their interaction. Ratings of EDI and 5D-ASC were analyzed using ANOVA with treatment as between group factor and ayahuasca experience as covariate. Pearson’s correlations were carried out to investigate the association between the ratings of ego dissolution and altered states of consciousness during the session and changes in outcome measures relative to baseline. Change scores from baseline were correlated with measures of EDI and the 5 key-dimensions of the 5D-ASC. The alpha criterion level of significant was set at *p* = .05.

## Results

### Study participants

There was no statistical difference in demographics (age, education, previous experience with ayahuasca, and other psychedelics) between groups. Participants (12 males, 18 females) had a mean (SD) age of 40.18 (10.10). Most participants were from Europe (*N*=28), while each one was from North America (*N*=1) and Asia (*N*=1). Furthermore, 11 participants held a bachelor’s degree, while the rest held a high school diploma (*N*=10), a master’s degree (*N*=2), or a PhD (*N*=2). All participants reported previous experience with ayahuasca. Overall, the participants had experienced ayahuasca 23.7 times (SD=15.58). Additionally, most participants (*N*=27) had previous experience with other substances (e.g., cannabis, LSD, psilocybin). Fourteen participants reported that they use alcohol, while 11 participants reported smoking, and 20 participants reported having a contemplative practice (e.g., meditation, yoga, prayer). Most participants (*N*=24) reported that they had relatives suffering from a mental disorder, but only one participant reported that the relative had a confirmed diagnosis of a mental health-related disorder and received treatment. Finally, common motivations of participants to ingest ayahuasca, besides partaking to become a facilitator, included understand myself (*N*=25), resolve problems (*N*=19), and curiosity (*N*=11). A total of 16 participants indicated that other motivations played an additional role as well, but these were not asked to be specified.

### Treatment guess

In the ayahuasca group, 8 out of 14 participants (57.1%) correctly guessed which condition they were assigned to, whereas the facilitators only guessed correctly in 5 out of 14 cases (38.5%). In the placebo group, 11 participants out of 16 (68.7%) guessed correctly which condition they were assigned to, and the facilitator guessed correctly in 14 out 16 cases (87.5%); see Table 1. Chi-square tests revealed no difference in the frequency of correct vs incorrect guesses among facilitators (*χ*^2^ (1)=3.57; *p*=0.06) and study participants (*χ*^2^ (1)=2.13; *p*=0.14). Likewise, there was no difference between the number of correct guesses by facilitators and study participants (*T*_27_ =0.81; *p*=0.42). However, guesses of facilitators and study participants were significantly correlated (*r*=0.54, *p*=0.003) suggesting some coherence between their correct and incorrect guesses.

### Subjective effects and MET

**Fig. 1:**
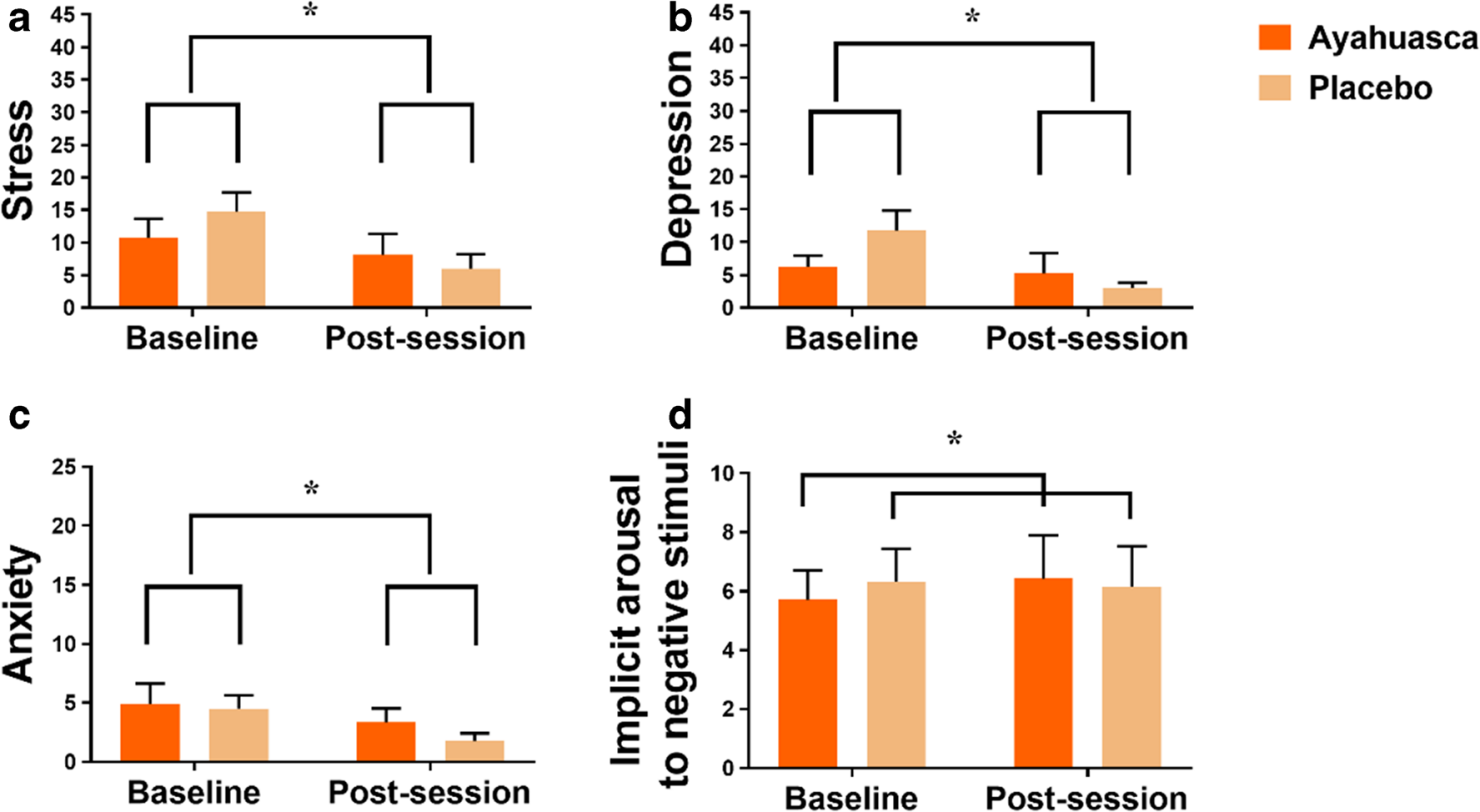
Mean ratings (SE) of stress (**a**) and depression (**b**), as assessed by DASS-21, as well as anxiety (**c**), as assessed by the BSI-18, and implicit arousal to negative stimuli (**d**), as assessed by MET. Statistical significance is indicated with an asterisk

Mixed model ANOVA revealed significant main effects of time on ratings of stress (*F*_1, 26_ = 8.27; *p* = .008, partial *η*2=0.24), depression (*F*_1, 26_ = 6.53; *p* = .017, partial *η*^2^=0.20) as assessed by the DASS-21, and on anxiety symptoms as assessed by BSI-18 (*F*_1, 26_ = 5.12; *p* = .032, partial *η*^2^=0.24). Mean (95% CI) change scores for these measures were −5.6 (−9.8 to −.15), −4.9 (−8.9 to −.73), and −2.1 (−2.4 to −.22), respectively. In addition, the interaction between treatment and time approached significance for the DASS-21 depression score (*F*_1, 26_ = 4.11; *p* = .053, partial *η*^2^=0.14), suggesting that the reduction in symptoms of depression was stronger in the placebo group. Overall, however, these findings indicate that ratings of stress, depression, and anxiety were lower after the ceremony as compared to baseline, independent of treatment group. Furthermore, a significant interaction between treatment and time was observed for the measure of implicit arousal to negative stimuli ratings on the MET (*F*_1, 16_ = 5.11; *p* = .038, partial *η*^2^=0.20), indicating that ayahuasca increased emotional empathy to negative stimuli, and placebo did not. Mean (SE) affect ratings and implicit arousal levels in both treatment groups are shown in Fig. 1. None of the FFMQ measures were affected by time or treatment. A summary of all statistical analyses is given in eTable 1 (supplement).

### The psychedelic experience

**Fig. 2:**
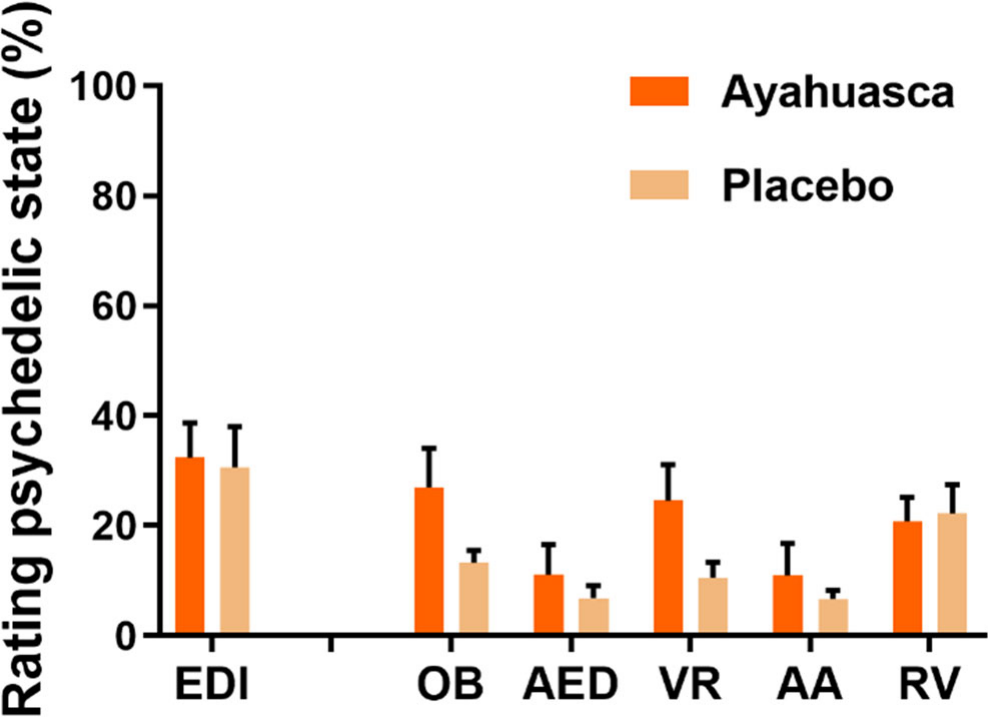
Mean ratings of the experience of altered states of consciousness as assessed by the EDI and 5D-ASC (from 0 to 100%) dimensions per group (ayahuasca and placebo). Abbreviations: Ego Dissolution Inventory (EDI), oceanic boundlessness (OB), anxious ego dissolution (AED), visual restructuralization (VR), auditory alterations (AA), and reduction of vigilance (RV)

There were no group differences between ratings of the total ego dissolution ratings. The overall mean (SD) rating of the experience of ego dissolution as assessed by the EDI was 32.39% (23.50) in the ayahuasca group and 30.66% (27.54) in the placebo group. Individual ratings of EDI are given in Table 1; mean EDI ratings are shown in Fig. 2.

Mean ratings on 5D-ASC dimensions varied between 10 and 27% in the ayahuasca group and between 6 and 23% in the placebo group. Furthermore, mean ratings on the 5D-ASC subscales varied between 11 and 34% in the ayahuasca group and 4 and 21% in the placebo group. Mean ratings of 5D-ASC dimensions are given in Fig. 2.

Mean ratings of EDI and total 5D-ACS (dimensions and subscales) did not significantly differ between conditions and did not significantly interact with ayahuasca use experience of the study participants (Supplement eTable 2). When Ayahuasca experience was removed as a covariate from the model, higher ratings in the ayahuasca group approached significance for oceanic boundlessness (*F*_1, 25_ = 3.54; *p* = .071), visual restructuralization (*F*_1, 25_ = 4.10; *p* = .054), experience of unity (*F*_1, 25_ = 3.55; *p* = .071), insightfulness (*F*_1, 25_ = 3.43; *p* = .076), and reached significance for insightfulness (*F*_1, 25_ = 5.86; *p* = .023). Mean ratings of EDI and total 5D-ASC did not differ between participants that received 7 or 10 capsules, in either treatment group.

## Correlational analysis

Correlations between change scores of depression, stress, anxiety, and emotional empathy (to negative stimuli) and EDI or 5D-ASC ratings failed to reach significance across the two groups. However, after a single outlier was removed (i.e. one study participant in the ayahuasca group showed strong increments in affect ratings after the ceremony) significant negative associations were found between changes in depression and ratings of anxious ego dissolution (r= -.59; p=.001) and auditory alterations (r= -.44; p=.026), and between changes in stress and ratings of anxious ego dissolution (r= -.42; p=.034). There was no correlation between number of previous ayahuasca experiences and the psychedelic experience as assessed with EDI and 5D-ASC.

## Discussion

The primary objective of the present study was to assess differences in responses to ayahuasca and placebo in participants of naturalistic ayahuasca ceremonies. In order to determine whether participants and ceremony facilitators were blind to the treatment randomization, we asked them to guess the treatment after the experience. Overall, 57.1% and 68.7% of the participants in the ayahuasca and placebo group, respectively, correctly guessed to which condition they were assigned. Facilitators guessed correctly in 38.5% and 87.5% of cases in the ayahuasca and placebo group, respectively. The frequency of correct and incorrect guesses did not significantly differ among facilitators and study participants, indicating that it was not overly evident for study participants and facilitators whether ayahuasca or placebo was assigned.

Mean subjective ratings of the psychedelic experience as assessed with the EDI and 5D-ASC were relatively low in the ayahuasca group as well as in the placebo group and did not markedly differ between groups. These findings contrast with previous research on ayahuasca which demonstrated that ingestion of the brew in a naturalistic setting induced a moderate experience of ego dissolution (Uthaug et al. 2018), possibly because alkaloid doses (DMT, harmine, harmaline) were relatively low in the present study. From the study of Uthaug et al. (2018), no information on the actual ayahuasca doses consumed is available, but mass spectrometry analyses of a number of 200-mL samples from the brew suggested the presence of moderate to high (i.e., 371–915 mg) DMT levels. In previous placebo-controlled studies, oral doses containing 0.36mg/kg DMT were administered to depressed patients (Palhano-Fontes et al. 2019) and freeze-dried oral doses containing 0.75mg/kg DMT (Dos Santos et al. 2012; Valle et al. 2016) and 1 mg/kg DMT (Dos Santos et al. 2011) to experienced users of ayahuasca. In the present study, doses were not adjusted for body weight. However, for an average individual of 70 kg, the equivalent dose would be between 0.20 (7 capsules) and 0.29 mg/kg (10 capsules). Therefore DMT doses in the present study were lower than a therapeutic dose of DMT as administered in a clinical setting. Moreover, depressed patients that were exposed to 0.36mg/kg DMT (Palhano-Fontes et al. 2019) were novice ayahuasca users whereas in the present study a similar dose was given to experienced ayahuasca users. The psychedelic experience of the depressed patients as rated with the Hallucinogenic Rating Scale and Mystical Experience Questionnaire achieved 20–60% of the maximal intensity which appears higher than psychedelic ratings in the present study that fluctuated between 10 and 30% of maximal intensity, albeit measured with different scales. Therefore, participants in the present study might have required a higher dose of DMT to achieve a stronger psychedelic experience, although the association between frequency of ayahuasca use and dosing requirement has not yet been established.

Another explanation for the low ratings of the psychedelic experience could be that participants lowered their expectancies because they were aware of the possibility that they may have been assigned to the placebo group which may have resulted in lowered ratings of the psychedelic experience (Colloca 2014). Conversely, ratings of the psychedelic experience of participants in the placebo group may have been boosted by their presence in a group ceremony in which most attendants drank ayahuasca and expressed their emotions and experiences (Olson et al. 2020). Together, ratings of the psychedelic experience in the present study indicate that participants in both groups experienced altered states of consciousness during the ceremony and that the strength of the mean experience was low, with individual experiences ranging from absent to strong.

Subjective ratings of symptoms of depression, stress, and anxiety were significantly less after the ceremony as compared to baseline, across both treatment groups. These positive changes did not differ between participants in the ayahuasca and placebo group, although decrements in symptoms of depression tended to be more prominent in the placebo group. This suggests an important role for non-pharmacological factors, such as set and setting. Set factors such as expectation, preparation, and intention can shape the response to hallucinogens (Hartogsohn 2014, 2017). Expectations are built from previous experience with the substance, and on general knowledge of its effects on affect and well-being (Haijen et al. 2018; Laska and Sunshine 1973; Metzner et al. 1965). Participants in the study had extensive previous experiences with ayahuasca and may have developed personal sets of expectation and intentions. Repeated participation in ayahuasca ceremonies might stimulate learned associations with enhanced well-being, which are memorized and experienced even when assigned to a placebo group. Similar mechanism have been proposed to explain the strength placebo effects in a wide range of medical patient groups (Colloca 2014; Haour 2005).

Additionally, it is known that expectancies are modeled through verbal suggestions and instructions (Bartels et al. 2014; Kirsch 1985, 2004; Martin-Pichora et al. 2011; Van Oorsouw and Merckelbach 2007). For example, (2007) it has been demonstrated that positive (“memory enhancing”) and negative (“memory impairing”) placebos may enhance and undermine, respectively, memory of a film fragment (Van Oorsouw and Merckelbach 2007). Specifically, it was found that in the positive placebo group, memory was better than that of participants who received negative placebos or control participants. Participants in the negative placebo group made more distortion errors than participants in the positive placebo or control group. In the context of the present study, one might speculate that (repeated) suggestion of the positive mental health effects of ayahuasca, by either peers or facilitator(s) throughout the ceremony, may have contributed to the positive changes in mental health parameters that were observed after the ceremony in both groups.

Likewise, the setting of the ceremony, such as the physical, social, and cultural environment, may alter the mental experience of a pharmacological agent (Hartogsohn 2014). Ceremonies included in the present study were always conducted in a supportive group environment which may very well have impacted the participants’ overall experience in a positive way and may have contributed to the improvements in affect (Adamson and Metzner 1988). Additionally, previous research has demonstrated that psychedelics, like LSD, can enhance suggestibility by temporarily suspending the drive to maintain control of one’s mind and environment (Carhart-Harris et al. 2015). This finding suggests that individuals can become unusually open and receptive to social group dynamics that take place during an ayahuasca ceremony and right after during integration sessions to support mental healing (Baker 2005). The latter however appeared not to have played a major role in the present study given the absence of a difference in nearly every dependent variable between the ayahuasca and placebo group. It should be noted, however, that the present study was not designed to distinguish the impact of set and setting on mental health outcomes from a moderating effect of ayahuasca on set and setting experience. To do so, a 2 (ayahuasca/placebo) × 2 (set and setting/no set and setting) design would be more appropriate. The present study primarily focused on the general impact of set and setting per se. In this context, it should also be noted that for many indigenous traditions, it is not necessary for the participants to consume ayahuasca. The belief held is that the shamans perform their work to aid those in the ceremony, even if they have not consumed the brew (Dos Santos and Hallak 2019).

The present findings do not mean that change in mental health outcomes following ayahuasca administration is always based on expectation and should always be qualified as a placebo effect. As noted in the introduction, there is strong evidence that ayahuasca can reduce symptoms of depression in treatment resistant patients as shown in a placebo controlled, randomized clinical trial (Palhano-Fontes et al. 2019). Likewise, subjective ratings of hopelessness and panic of Santo Daime members decreased 1 h after ayahuasca use as compared to placebo (Santos et al. 2007). Also the present study provided evidence for a pharmacological induced change in mental state. Participants that were assigned to the ayahuasca group displayed a significant increase in arousal to negative emotions that was not observed in the placebo group. This increase in empathic emotion was assessed with the MET that might be less susceptible to the influence of non-pharmacological factors and fluctuates with drug concentration (Kuypers et al. 2017b). Similar findings have been reported in naturalistic studies on other psychedelics such as psilocybin, albeit in the absence of a placebo control group (Mason et al. 2019). Overall, the present finding is important as low-level empathy has been found in stress-related psychopathologies like depression, anxiety disorders, and post-traumatic stress disorder (PTSD) (Chamberlain et al. 2006; Cusi et al. 2011; Donges et al. 2005; Lee and Orsillo 2014; Morrison et al. 2016; Nietlisbach et al. 2010; Palm and Follette 2011; Parlar et al. 2014). Treatments that increase empathy may be very relevant for patients that suffer from mood disorders and psychopathy. Core features of mood disorders include repetitive and rigid patterns of negative and compulsive thoughts, together with social difficulties and impaired empathic abilities (Aldao et al. 2010; Beck 1967; Dos Santos et al. 2016; Morrison et al. 2016; Nietlisbach and Maercker 2009; Todd et al. 2015). The lack of empathy is particularly evident in psychopathy and has been suggested to modulate an individual’s risk for aggression (Blair 2018; Cusi et al. 2011; Decety et al. 2013; Donges et al. 2005).

Negative associations were found between 5D-ASC ratings and changes in affect rating across the two treatment groups. This suggests that the magnitude of symptom reduction is related to the strength of the psychedelic experience, i.e., the stronger the experience, the more prominent the reduction in symptoms. Previous studies in naturalistic settings have reported similar associations between strength of the psychedelic experience and the magnitude of subjective mental health changes following the use of ayahuasca (Palhano-Fontes et al. 2019; Uthaug et al. 2018) and 5-MeO-DMT (Uthaug et al. 2019). As the psychedelic experience did not greatly differ in magnitude between the ayahuasca and the placebo group, the present study additionally suggests that the association between the magnitude of the psychedelic experience and magnitude mental health benefits prevails independent of the means (i.e., a psychedelic or placebo) through which the psychedelic state was actually achieved. This finding further attest to the notion that also a placebo response can elicit significant clinical benefits on mental health outcomes (Dodd et al. 2015; Mitsikostas et al. 2014).

The present study has limitations. Participants were very experienced users of ayahuasca which makes it rather likely that expectancy effects contributed strongly to outcome measures in both groups. Expectancy effects may be less in novel ayahuasca users, who therefore may be more susceptible to the pharmacological impacts of ayahuasca. Future studies should also investigate the impact of ayahuasca and set and setting on the ayahuasca experience of novel users and include a priori measures expectation in their study designs. It should also be noted that the investigators were not in control of set and setting during ceremonies which makes it impossible to single out specific set and setting parameters that contributed to changes in mental health outcomes observed in the study participants. Likewise, the investigators were not in control of the ayahuasca administration, preparation, and storage, and no information on the stability of the ayahuasca capsules throughout the course of the study was available. Mean subjective ratings of the overall psychedelic experience as assessed with the 5D-ASC and EDI centered around 10–30% of the maximal score, suggesting that the dose may have been too low to elicit a full-blown psychedelic response. It therefore cannot be excluded that a distinction between pharmacological and non-pharmacological contribution to changes in mental health outcomes would have become more prominent at higher doses of ayahuasca.

In sum, the current findings demonstrate that improvements in mental health of participants in naturalistic ayahuasca ceremonies can be driven by non-pharmacological factors that elicit a placebo response but also by pharmacological factors that are related to the use of ayahuasca. The present findings warrant further research into the non-pharmacological factors contributing to the mental health effects following ingestion of ayahuasca as well as other psychedelics ingested during group ceremonies.

## Supplementary Information

ESM 1 (DOCX 18 kb)
