# Supplementary material for: A placebo-controlled study of the effects of ayahuasca, set and setting on mental health of participants in ayahuasca group retreats
Source: Psychopharmacology (Berl). 2021 Mar 10;238(7):1899–910. doi: 10.1007/s00213-021-05817-8 (PMC8233273; doi:10.1007/s00213-021-05817-8)
Supplement: Supplementary file 1 — (DOCX 18 kb) [file 213_2021_5817_MOESM1_ESM.docx]

**Supplemental information**

**eTable 1. Mean (SE) scores per treatment group for all dependent variables and mixed-model ANOVA results.**

| **Variable** | **Time** | **Placebo**  **group** | **Ayahuasca group** | **Df** | **Treatment** | **Time** | **Treatment x Time** |
| --- | --- | --- | --- | --- | --- | --- | --- |
| *Dass-21* |  | Mean (se) | Mean (se) |  | F; p | F, p | F, p |
| Stress | Pre | 14.71 (3.00) | 10.71 (2.93) | 1,26 | 0.06; 0.8 | 8.27; **0.008** | 2.45; 0.129 |
|  | Post | 6.0 (2.26) | 8.14 (3.15) |  |  |  |  |
| Depression | Pre | 11.71 (3.10) | 6.28 (1.70) | 1,26 | 0.45; 0.51 | 6.53; **0.017** | 4.11; 0.053 |
|  | Post | 3.00 (0.08) | 5.28 (2.45) |  |  |  |  |
| Anxiety | Pre | 8.12 (2.34) | 5.71 (2.84) | 1,26 | 0.20; 0.89 | 2.68; 0.11 | 0.93; 0.34 |
|  | Post | 2.57 (1.05) | 4.6 (2.57) |  |  |  |  |
| *BS1-18* |  |  |  |  |  |  |  |
| Depression | Pre | 5.14 (1.13) | 2.85 (0.91) | 1,26 | 0.95; 0.34 | 1.63; 0.21 | 1.31; 0.26 |
|  | Post | 2.50 (0.65) | 2.71 (1.34) |  |  |  |  |
| Somatization | Pre | 3.14 (1.09) | 3.71 (1.20) | 1,26 | 0.07; 0.78 | 0.05; 0.81 | 0.05; 0.81 |
|  | Post | 3.14 (1.30) | 3.53 (0,86) |  |  |  |  |
| Anxiety | Pre | 4.50 (1.13) | 4.92 (1.71) | 1,26 | 0.43; 0.51 | 5.12; **0.032** | 0.36; 0.55 |
|  | Post | 1.78 (0.69) | 3.35 (1.19) |  |  |  |  |
| *FFMQ-15* |  |  |  |  |  |  |  |
| Observe | Pre | 11.96 (0.66) | 12.76 (0.42) | 1,25 | 2.57; 0.12 | 1.07; 0.31 | 0.67; 0.41 |
|  | Post | 11.14 ( 0.65) | 12.15 (0.56 ) |  |  |  |  |
| Describe | Pre | 10.85 (0.82) | 10.76 (0.72) | 1,25 | 1.78 ;0.19 | 0.10; 0.75 | 0.47; 0.49 |
|  | Post | 11.50 (0.80) | 11.53 (0.91) |  |  |  |  |
| Awareness | Pre | 10.85 (0.81) | 10.76 (0.64) | 1,25 | 0.01; 0.97 | 2.08; 0.16 | 0.02; 0.89 |
|  | Post | 11.50 (0.70) | 11.53 (0.73) |  |  |  |  |
| Non-judgement | Pre | 11.64 (0.80) | 12.23 (0.73) | 1,25 | 0.11; 0.74 | 2.29; 0.14 | 0.26; 0.61 |
|  | Post | 12.57 (0.64) | 12.69 (0.88) |  |  |  |  |
| Non-reaction | Pre | 9.21 (0.62) | 10.15 (0.75) | 1,25 | 1.08; 0.31 | 0.01; 0.95 | 0.04; 0.84 |
|  | Post | 9.35 (0.82) | 10.07 (0.72) |  |  |  |  |
| *MET* |  |  |  |  |  |  |  |
| IEE positive | Pre | 5.03 (1.85) | 4.73 (1.67) | 1,16 | 0.01; 0.97 | 2.44; 0.14 | 0.96; 0.34 |
| IEE positive | Post | 5.22 (1.80) | 5.56 (1.98) |  |  |  |  |
| IEE negative | Pre | 6.33 (1.08) | 5.71 (1.05) | 1,16 | 0.09; 0.76 | 1.99; 0.17 | 5.11; **0.038** |
| IEE negative | Post | 6.16 (1.36) | 6.43 (1.46) |  |  |  |  |
| EEE positive | Pre | 3.71 (2.05) | 3.36 (2.07) | 1,16 | 0.20; 0.65 | 2.15; 0.16 | 1.85; 0.19 |
| EEE positive | Post | 3.77 (1.72) | 4.93 (2.76) |  |  |  |  |
| EEE negative | Pre | 6.41 (1.04) | 5.86 (0.99) | 1,16 | 0.25; 0.62 | 2.48; 0.14 | 3.48; 0.08 |
| EEE negative | Post | 6.37 (1.18) | 6.39 (1.28) |  |  |  |  |

**eTable 2. Mean (SE) ratings of the EDI and 5D-ASC dimensions and subscales in each treatment group, and ANOVA of the factor Treatment and the interaction of Treatment with the covariate ‘Experience in ayahuasca use’**

| **Variable** | **Ayahuasca** | **Placebo** | **Treatment** | | **Treatment x Ayahuasca Experience** | |
| --- | --- | --- | --- | --- | --- | --- |
|  | **Mean (SE)** | **Mean (SE)** | **F_(1,23)_** | **p** | **F_(2,23)_** | **p** |
| *EDI* |  |  |  |  |  |  |
| EDI | 32.39 (6.28) | 30.66 (7.36) | 0.01 | 0.92 | 0.59 | 0.55 |
| *5D-ASC* |  |  |  |  |  |  |
| Oceanic boundlessness | 26.97 (7.18) | 13.24 (2.24) | 0.87 | 0.71 | 0.37 | 0.69 |
| Anxious ego dissociation | 11.06 (5.45) | 6.76 (2.30) | 1.41 | 0.24 | 1.76 | 0.19 |
| Visual restructuralization | 24.56 (6.56) | 10.48 (2.79) | 1.31 | 0.26 | 0.31 | 0.74 |
| Auditory alterations | 10.85 (5.86) | 6.63 (1.60) | 1.42 | 0.25 | 2.46 | 0.11 |
| Reduction of vigilance | 20.77 (4.33) | 22.21 (5.22) | 0.51 | 0.48 | 1.28 | 0.29 |
|  |  |  |  |  |  |  |
| Experience of unity | 28.88 (8.21) | 13.48 (3.50) | 0.12 | 0.72 | 0.32 | 0.73 |
| Spiritual experience | 24.25 (7.28) | 13.69 (3.66) | 0.01 | 0.90 | 0.26 | 0.77 |
| Blissful state | 28.28 (7.75) | 20.83 (3.91) | 1.60 | 0.21 | 2.45 | 0.11 |
| Insightfulness | 33.44 (9.38) | 10.59 (2.61) | 0.19 | 0.66 | 0.90 | 0.42 |
| Disembodiment | 16.21 (6.87) | 5.80 (1.65) | 1.5 | 0.22 | 0.19 | 0.82 |
| Impaired control and cognition | 11.54 (6.29) | 8.30 (3.23) | 0.45 | 0.51 | 1.17 | 0.32 |
| Anxiety | 12.40 (6.11) | 4.57 (1.46) | 4.15 | 0.06 | 2.40 | 0.19 |
| Complex imagery | 30.15 (9.30) | 12.40 (3.29) | 0.13 | 0.71 | 0.28 | 0.76 |
| Elementary imagery | 26.36 (7.72) | 12.20 (4.01) | 2.76 | 0.11 | 1.60 | 0.22 |
| Audio visual synesthesia | 23.18 (7.99) | 9.00 (4.53) | 5.85 | 0.02 | 4.33 | 0.03 |
| Changed meaning of perception | 27.19 (7.93) | 12.87 (4.23) | 0.49 | 0.48 | 0.11 | 0.90 |
